# The Centennial of the Discovery of Chagas Disease: Facing the Current Challenges

**DOI:** 10.1371/journal.pntd.0000645

**Published:** 2010-06-29

**Authors:** Joseli Lannes-Vieira, Tania C. de Araújo-Jorge, Maria de Nazaré Correia Soeiro, Paulo Gadelha, Rodrigo Corrêa-Oliveira

**Affiliations:** 1 Fiocruz Program for Research and Technological Development on Chagas Disease (PIDC) Fundação Oswaldo Cruz, Rio de Janeiro, Brazil; 2 Fundação Oswaldo Cruz, Rio de Janeiro, Brazil; National Institutes of Health, United States of America

## Chagas Disease: Then and Now

One hundred years after Carlos Chagas' discovery [1] WHO defines Chagas disease as one of the most important infectious diseases of poverty. Beyond its biological determinants (interplays among the parasite, vector, and human), the social determinants of Chagas disease are of utmost importance; poor housing and working conditions, low salaries, and malnutrition are directly linked to Chagas disease in Latin American [2]. This article highlights the current state on research and innovation related to control and care of Chagas disease, as well as the challenges for the next decade.

## Confronting Reality and Keeping Transmission Control in Focus

In 2007, Latin America [3] had an estimated 8 million to 15 million *Trypanosoma cruzi*-infected individuals in 15 afflicted countries, with an incidence of vector transmission greater than 40,000 cases per year and the congenital transmission rate higher than 14,000 cases per year. These numbers reflect approximately 2 million infected women of fertile age and almost 30 million people at risk in endemic areas. The prevalence of infected blood in blood banks is 1.28%, and 1.8 million individuals have Chagas disease cardiopathy [4], with several hundred thousand infected people living in other nations as a result of migration [5].

Considering the feasibility of Chagas disease control, several points must be discussed, namely:

- Permanent surveillance for new outbreaks due to oral transmission;
- Domiciliary invasion by sylvatic triatomines, due to the complex parasite behaviour and ecological contexts;
- Lack of effective treatment, immunoprophylaxis, and feasible markers for disease progression; and
- Continuous circulation of *T. cruzi* isolates largely spread in sylvatic ecotopes across the American continent, showing that elimination of human disease transmission has to be seen in a new context [6] that is dependent on local transmission characteristics and prevalence [4], [5].

Epidemiological parameters have shown that the success of vector control policies are due to efforts of large interventions such as those in the Southern Cone, Andes, Central America, Mexico, and Amazon, which have largely reduced the morbidity and transmission of Chagas disease [3], [4]. In Brazil, for example, the success of vector control reduced the incidence of new cases from 100,000 in 1980 to less than 500 notified cases per year from 2001 to 2006 [7]. Uruguay, Chile, and Brazil received the Pan American Health Organization (PAHO)/World Health Organization (WHO) certification for interruption of *Triatoma infestans* vector transmission [3]. However, sustainability depends on control of autochthonous triatomines by permanent epidemiological and entomological surveillance, as annual oscillating frequencies of house infestation within the same region have been noted, particularly in some hotspots in the Argentinean Gran Chaco [8]. Limited effectiveness of pyrethroid insecticides outdoors and in peridomiciliar structures, development of insecticide resistance, and sylvatic and semi-domestic vectors might contribute to residual vector foci even after insecticide spraying [8], implying a risk of re-emergence of acute Chagas disease. Additionally, blood bank control still reveals high frequencies of seropositive donors and more attention should be given to the frequency of seropositive pregnant women and congenital transmission, when early access to diagnosis and treatment is crucial and should be mandatory [6], [7]. Together with the success of the control programs in some countries of the Southern Cone, progress in other endemic areas has been uneven. Maintenance of previously successful control programs as in Venezuela, fighting to increase economic resources as in Central America, and strengthening of official control programs in Mexico and Peru would be important to extend the success of southern countries to all endemic areas.

An important issue that has evolved in current programs was the divorce of vector control from Chagas disease assistance to chronic patients [9]. The first is field-based with routine insecticide spraying and vector surveillance; the second is hospital-based in parallel to blood bank screening, and diagnosis with treatment and medical care. More integrated Chagas disease control and patient management would combine vector control with case detection/treatment and broad social participation. This strategy might help overcome the difficulties and increase cost-effectiveness of the control program, its public acceptance, and its long-term sustainability. It is important to educate the community for the surveillance of vector populations, to create more explicit guidelines for basic health systems, and to rapidly detect new acute cases in order to avoid the re-emergence of Chagas disease. Among the challenges to be addressed in the next decades is the continuous maintenance of epidemiological surveillance, which is cost/benefit–favourable. However, its priority is low, since Chagas disease afflicts mostly the poor and socially marginalized populations, with programs performed by the public sector, implying political decisions in sometimes unstable conditions. In this scenario, new actors emerge: associations of patients in affected countries and non-governmental organizations (NGOs), such as the Drugs for Neglected Diseases *initiative* (DND*i*) and Médicins Sans Frontières (MSF). Community engagement and motivation as well as integration of prevention, including educational strategies, and treatment are huge compromises that must be made, and a “task shift” must occur to mitigate a human resource crisis.

## A Vaccine for Chagas Disease: Reality or Fantasy?

Almost 25 years ago it was believed that a vaccine against *T. cruzi* would fulfil the primary requirements of any proposed vaccine, such as *primum non nocere* (first, do no harm), inducing sterile immunity, and being effective against all strains. However, the autoimmune hypothesis related to Chagas disease pathogenesis hampered efforts to develop new chemotherapy and vaccine approaches against *T. cruzi*. Later, the demonstration that parasite persistence was associated with Chagas disease progression emphasized the importance of parasite control in ameliorating or hampering disease evolution [10]. Therefore, vaccines and new antiparasite drugs became realistic proposals. It is still unclear whether a prophylactic vaccine has a place in the current epidemiological picture of Chagas disease control, whether it would be better as an immunotherapeutic agent to delay disease progression, or whether it should be developed for dogs, which may play a role as domestic and peridomestic reservoirs [11]. Furthermore, immunotherapeutic vaccines may be alternatives for improving prognosis in chronic patients. Evidently, with success will come the economic barriers to produce and deliver the vaccine, a common feature of neglected diseases.

## Chagas Disease Therapy: Exploring the Knowledge Frontier

For more than three decades, only two drugs have been available for Chagas disease: nifurtimox and benznidazole [12]. Although their use in the acute and early chronic phases of Chagas disease can reach a 70% cure rate, solid scientific evidence is still lacking about its use in the chronic phase. The BENEFIT program, a multi-centric and non-randomized follow-up to evaluate the effect of benznidazole in chronic patients is underway [12]. It is one among the only five clinical trials for Chagas disease registered in the Clinical Trials database (http://www.clinicaltrials.gov), a clear indication of the negligence of the pharmaceutical industry. The urgently needed pediatric formulation for benznidazole has fortunately been undertaken by a public pharmaceutical company in Brazil (LAFEPE) and DND*i*.

Two main approaches to the development of new drugs against Chagas disease have been used: target-based strategy, requiring the study of *T. cruzi* biology to discover and target a weak link in metabolism or pathogenesis; and diversity or phenotypic screens—new uses for existing drugs [13]. In fact, sequencing the *T. cruzi* genome [14] was an important advance in understanding its biology and in improving the identification of possible new drug targets. Re-annotation and metabolic pathway reconstruction helps to predict enzymatic functions and characterize some unique enzymes and pathways in *T. cruzi* aiming to recognize new potential targets. Other approaches have also advanced the process: ergosterol biosynthesis inhibitors, potent anti-fungals, and promising anti-trypanosomatid agents that are nearing clinical trials for Chagas disease, such as ravuconazole and posaconazole [15].

Another advance includes the combination of registered drugs (nifurtimox and benznidazole) and their combinations with other trypanocide compounds and/or other drugs as amiodarone, an anti-arrhythmic agent that also has anti-*T. cruzi* activity, acting synergistically with posaconazole [16]. Cell therapy in animal models and in humans have shown improvement in right ventricular alterations, although their long-term analysis revealed that the beneficial effects were not long lasting [17]. A new avenue to be explored involves the use of immunomodulators alone or in association with trypanocidal drugs to ameliorate the chronic effects of *T. cruzi* infection.

## Diagnosis and Treatment Follow-up: Challenges in Chagas Disease

Significant progress has been achieved in diagnosis since the use of xenodiagnosis and serologic tests to confirm Chagas disease. However, although reliable serology assays are currently available, especially for chronic cases, cure and adequate follow-up diagnostic methods remain a challenge [18]. After benznidazole therapy of chronic patients, conventional serology may remain positive for several years despite repeated negative parasite detection [18]. New tools are still necessary and several new tests need to be validated.

The use of PCR to detect minimal numbers of *T. cruzi* DNA in the blood samples has opened new possibilities for the diagnosis of acute and chronic Chagas disease and as an adjunct to conventional serology [19]. Diagnosis in the Amazon region (Peru, Ecuador, and Brazil) remains a puzzle and serology requires appropriate antigens to represent the heterogeneity of the *T. cruzi* strains. It is generally agreed that evidence is lacking that *T. cruzi* is more than a single species; although the geographical distribution of *T. cruzi* I and *T. cruzi* II in humans varies, both induce disease [20]. Studies with microsatellites and mtDNA sequence variants produced evidence for the existence of a third phylogenetic *T. cruzi* lineage, named *T. cruzi* III [21]. The questions of whether or not triatomines play a role as “biological filters” and whether or not the immune system differentially controls *T. cruzi* lineages await further exploration. A new multiplex assay platform designed to detect anti-*T. cruzi* antibodies using recombinant antigens and parasite lysate may be useful to improve sensitivity and specificity in diagnostic tests [22]. In addition, the variety and quality of diagnostic targets for Chagas disease have been substantially improved, offering a useful tool for determining treatment success or failure [23]. Furthermore, the recognition of parasite-specific immune response alterations after benznidazole treatment during chronic Chagas disease has raised the possibility of development of biomarkers indicative of treatment efficacy and cure [24].

## Chagas Disease: What Can Be Done to Care for the Patients?

Chagas heart disease is the leading cause of infectious myocarditis worldwide, and is the most serious and frequent manifestation of chronic Chagas disease, with 20%–40% of infected individuals affected 10–30 years after infection. Persistent low-grade parasitism accompanied by an unbalanced immunological response seems to be the main pathogenic mechanism that leads to myocardial damage, together with microvascular dysfunction and neuronal loss [10]. Currently, patient management only mitigates the main symptoms [9]. The establishment of risk stratification models and algorithms to guide mortality risk assessment for therapeutic decision-making are crucial, as is evaluation of functional heart impairment and prognosis to improve quality of life and survival rates [10], [25]. Multidisciplinary teams committed to the care of Chagas disease patients have shown that a broader vision of health care might help clinicians make decisions closer to the daily reality of the patient and have a positive impact on treatment adherence and individual quality of life [9], [25]. The consensus on priorities for Chagas disease indicates the need for:

- Holistic care of the patients beyond solely conventional therapy;
- Innovation on drugs or combined therapies that provide a shorter treatment course with fewer side effects, and on paediatric formulations;
- New tools to follow up treatment efficacy during the chronic phase; and
- New biomarkers to identify cure and assist with stratification and prognosis of risk factors.

## Keep Holding On

**Figure 1:**
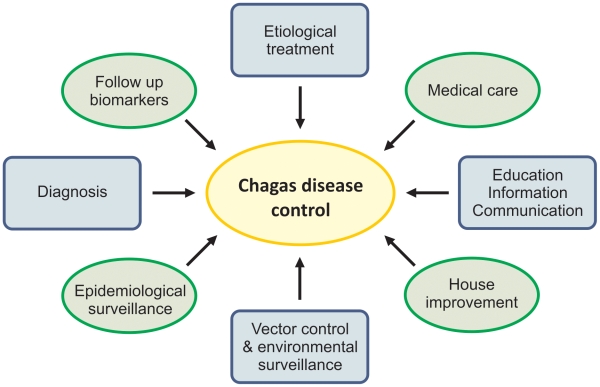
Chagas disease control: Hubs, challenges, and opportunities for the next decade.

Crossing the “valley of death” [26]—the enormous gap between discovery/basic science and health products and services—is a current and permanent challenge. Translational research has become a major agenda issue [27] and is certainly needed for all diseases including those poor populations that suffer the “neglected diseases.” Regarding Chagas disease, recognition of the relevant decrease in the number of new acute cases in most countries due to successful vector control programs in the last decades is certainly not sufficient to control the disease. As shown in Figure 1, current social and epidemiological scenarios point to the following needs:

- Sustainable and integrated surveillance strategies and identification of priority areas for immediate intervention;
- Provision of adequate, universal, integral, and holistic care to patients, with access to available and new therapies;
- Improvement of diagnostic and prognostic markers for disease progress, aiming for more rational interventions;
- Guaranteed access to information, education, and social organisations for Chagas disease patients and their families;
- Creation of more organized, integrated, and collaborative networks between researchers, physicians, patients, and policy makers.

A forum is urgently needed to address the challenges of keeping Chagas disease on the agendas of scientific and funding agencies. Even a hundred years after, Carlos Chagas' thoughts are undoubtedly valid right now: “…for Chagas, only science directed towards the betterment of humanity was valid (…) Thus he never forgot that social and economic factors are responsible—to a very great extent—for the situations he witnessed. In this way he was a precursor to what is most noble about social medicine. In this respect it is important to remember his words about the elimination of American trypanosomiasis (…), that the construction of decent housing, compatible with healthy way of life, was the most necessary element” [28].
